# The Combined Quantification and Interpretation of Multiple Quantitative Magnetic Resonance Imaging Metrics Enlightens Longitudinal Changes Compatible with Brain Repair in Relapsing-Remitting Multiple Sclerosis Patients

**DOI:** 10.3389/fneur.2017.00506

**Published:** 2017-09-27

**Authors:** Guillaume Bonnier, Benedicte Maréchal, Mário João Fartaria, Pavel Falkowskiy, José P. Marques, Samanta Simioni, Myriam Schluep, Renaud Du Pasquier, Jean-Philippe Thiran, Gunnar Krueger, Cristina Granziera

**Affiliations:** ^1^A.A. Martinos Center for Biomedical Imaging, Massachusetts General Hospital, Harvard Medical School, Charlestown, MA, United States; ^2^Advanced Clinical Imaging Technology (HC CMEA SUI DI BM PI), Siemens Healthcare, Lausanne, Switzerland; ^3^Signal Processing Laboratory 5 LTS5, École Polytechnique Fédérale de Lausanne, Lausanne, Switzerland; ^4^Donders Centre for Cognitive Neuroimaging, Radbound University, Nijmegen, Netherlands; ^5^Neuropsychology, Institution de Lavigny, Denens, Switzerland; ^6^Neurology Service and Neuroimmunology Laboratory, Department of Clinical Neurosciences, Centre Hospitalier Universitaire Vaudois, University of Lausanne, Lausanne, Switzerland; ^7^Siemens Medical Solutions USA IM MR COL NEZ, Burlington, MA, United States

**Keywords:** magnetization transfer imaging, relaxometry, advanced magnetic resonance imaging techniques, multiple sclerosis, relapsing-remitting, brain repair

## Abstract

**Objective:**

Quantitative and semi-quantitative MRI (qMRI) metrics provide complementary specificity and differential sensitivity to pathological brain changes compatible with brain inflammation, degeneration, and repair. Moreover, advanced magnetic resonance imaging (MRI) metrics with overlapping elements amplify the true tissue-related information and limit measurement noise. In this work, we combined multiple advanced MRI parameters to assess focal and diffuse brain changes over 2 years in a group of early-stage relapsing-remitting MS patients.

**Methods:**

Thirty relapsing-remitting MS patients with less than 5 years disease duration and nine healthy subjects underwent 3T MRI at baseline and after 2 years including T1, T2, T2* relaxometry, and magnetization transfer imaging. To assess longitudinal changes in normal-appearing (NA) tissue and lesions, we used analyses of variance and Bonferroni correction for multiple comparisons. Multivariate linear regression was used to assess the correlation between clinical outcome and multiparametric MRI changes in lesions and NA tissue.

**Results:**

In patients, we measured a significant longitudinal decrease of mean T2 relaxation times in NA white matter (*p* = 0.005) and a decrease of T1 relaxation times in the pallidum (*p* < 0.05), which are compatible with edema reabsorption and/or iron deposition. No longitudinal changes in qMRI metrics were observed in controls. In MS lesions, we measured a decrease in T1 relaxation time (*p*-value < 2.2e−16) and a significant increase in MTR (*p*-value < 1e−6), suggesting repair mechanisms, such as remyelination, increased axonal density, and/or a gliosis. Last, the evolution of advanced MRI metrics—and not changes in lesions or brain volume—were correlated to motor and cognitive tests scores evolution (Adj-*R*^2^ > 0.4, *p* < 0.05). In summary, the combination of multiple advanced MRI provided evidence of changes compatible with focal and diffuse brain repair at early MS stages as suggested by histopathological studies.

## Introduction

Relapsing-remitting multiple sclerosis (RRMS) is a chronic progressive disease that evolves through clinical relapses and subclinical inflammation and degeneration (1). To date, there is no cure for RRMS but a number of treatments substantially reduce the frequency of clinical relapses and the extent of local inflammation and brain volume loss (2). Conventional magnetic resonance imaging (cMRI), such as T1- and T2-weighted magnetic resonance imaging (MRI), provides essential metrics to measure local pathology (lesion number and volume), inflammatory activity, and severe tissue loss in the central nervous system (3). Yet, cMRI measures exhibit low sensitivity to diffuse brain alterations, do not provide metrics to differentiate inflammatory demyelination from brain remodeling and repair (4), and only partially correlate with patients function and disability (5). To overcome the limitations of cMRI, a number of non-conventional MRI techniques have been studied in MS patients, including Magnetization Transfer Imaging (MTI), Diffusion Tensor Imaging Susceptibility MRI, and MR relaxometry (5).

Among non-conventional MRI techniques, quantitative MRI (qMRI) probes the brain microstructure through standardized physical parameters that are directly linked to the biological and/or pathological properties of the tissue where they are measured (6). Besides, the combination of multiple qMRI metrics allow to amplify the true tissue-related information and limit their inherent measurement noise, thanks to the overlapping information that qMRI provide (7, 8). In fact, qMRI contrasts are influenced by the same tissue properties (i.e., micro/macromolecules, i.e., within axons, myelin, cells, intracellular/extracellular water, iron), though they exhibit different sensitivity to each of them (6, 9, 10). T1 relaxometry is highly sensitive to the tissue structural organization and free-water protons (11, 12): T1 rt increases when there is a loss of tissue structural organization/density (13, 14), and/or when free-water content increases. T1 rt also moderately decreases with an increase of iron content in the brain tissue (15). T2 rt is highly sensitive to free-water content, although it is also affected by the presence of macromolecules and iron. Both an accumulation of free water and a loss of macromolecules will increase T2 rt, while iron will decrease it (16). As well, the T2* transverse rt exhibit high sensitivity to free-water content and it is particularly sensitive to the presence of iron (17). An increase of T2* suggests a loss of macromolecules, while a decrease indicates an increase of iron or macromolecular compounds. Finally, the metrics obtained with MTI are very sensitive to variation of large molecular aggregates like lipids and proteins in myelin or cellular membranes (18, 19) so that they decrease when the amount of macromolecules decreases (i.e., in demyelination and/or cell loss) and/or the free water increases (i.e., edema) (20). Specific patterns of changes in T1, T2, T2* relaxation times, and magnetization transfer measures may be optimal to quantify brain abnormalities and their evolution in MS patients. For example, an increase in T1 and T2 relaxation times may suggest either a water accumulation (edema) or a loss of myelin/axon. A concomitant strong decrease in MTR will point at the latter phenomenon (myelin/axon loss), whereas a mild MTR decrease or no MTR changes are more typical of an increase in extracellular water (6). Combining multiple qMRI techniques in a clinically compatible protocol is challenging (e.g., due to motion, scan time, reproducibility, etc.) but there are currently ongoing efforts toward fast and reproducible protocols applying combined and accelerated acquisitions (21–23), synthetic computation of qMRI metrics (22) and qMRI fingerprinting (24).

To date, a number of cross-sectional studies [for review, see Ref. (5, 25)]—including some from our group (26–28)—leveraged the information obtained by multiple qMRI contrasts to describe brain inflammation and degeneration in MS patients. However, only few applied this approach to study longitudinal brain changes in patients with MS (29, 30) and reported the concomitant changes of multiple qMRI metrics [myelin water fraction, MTR, T1, and T2 relaxometry (29) and MTR and fractional anisotropy/diffusivities (30)] in black-holes and brain connectivity.

In this work, we have developed an automated MRI framework combining T1, T2, and T2* relaxometry with MTI (hereafter referred as qMRI) in order to (i) assess the potential of a combination of multiple qMRI parameters to quantify and interpret the evolution over 2 years of focal and diffuse pathology in a cohort of early RRMS patients and (ii) predict patients clinical outcome.

## Materials and Methods

### Population and Clinical Assessment

We enrolled 36 RRMS patients and 18 HC who had a baseline MRI study and 30 RRMS patients and 9 HC came back for a MRI follow-up study at 2 years ± 1 week after baseline. Data from seven patients were discarded because of (i) artifacts such as motion or ringing, which were identified by visual inspection in one or more datasets or (ii) because of missing data. Cognitive assessment was performed in patients at both time-points and in HC at baseline.

Inclusion criteria for patients were definite MS diagnosis according to the revised diagnostic criteria (31), less than 5 years disease duration at enrollment, age comprised between 20 and 70 years old and no other neurological or psychiatric diseases. For healthy controls, inclusion criteria were the absence of neurological, psychiatric, or systemic disease and age between 20 and 70 years old. Table 1 reports the population demographics and clinical characteristics. Eighty-seven percent of the patients were under immunomodulatory treatment (high-dosage IFN beta or fingolimod) for at least 3 months at the first time-point (TP1) and 97% at 2-year follow-up (TP2). Treated patients remained on the same treatment for the entire duration of the study. No patient had received corticosteroid therapy within the 3 months preceding the enrollment and follow-up MRI.

**Table 1:** Population demographics.

Demographics	RRMS (*n* = 23)	HC (*n* = 9)	RRMS vs HC
Age (years) (SD)	35.7 (11.8)	34.3 (9.2)	ns
Gender (male/female)	8/15	5/4	ns
Time since first relapse (months) (SD)	33.2 (22.4)	N.A.	N.A.
Subjects under treatment	20 (87%)	N.A.	N.A.
Time since immunomodulatory^a^ treatment (months)	>3	N.A.	N.A.

*RRMS, relapsing-remitting MS patients; HC, healthy controls*.

*^a^High-dosage interferon beta and fingolimod*.

The study was approved by the Ethic committee of the Lausanne University Hospital (CHUV). All participants gave written informed consent prior to enrollment.

### Clinical Assessment

At both time-points, each subject underwent neurocognitive, behavioral, motor, and disability examination by a certified neurologist (Cristina Granziera), as specified in Table 2. To reduce training effects, we administered one different version per time-point out of the two parallel batteries available for the Brief Repeatable Battery of Neuropsychological Tests (BRB-N) (32).

**Table 2:** Clinical scores.

Disability, motor, and cognitive tests		Function	Patients scores at TP1	Patients scores at TP2	Controls scores at TP1	Patients vs controls for non-continuous scores	
						TP1	TP2
BRB-N cognition	SRT-LTS	Verbal memory	62.5 ± 6.6 (−0.3)	66.1 ± 5 (0.21)	65.1 ± 6.8	N/A	N/A
	SRT-CLTR	Verbal memory	57.7 ± 10.4 (−0.14)	62.4 ± 9.6 (0.26)	60.1 ± 10	N/A	N/A
	SRT-recall	Verbal memory	11.2 ± 1.2 (−0.03)	11.7 ± 0.4 (0.11)	11.6 ± 0.9	N/A	N/A
	SRT-delayed recall	Verbal memory	8.1 ± 2.5 (0.11)	8.3 ± 2.5 (0.04)	8.4 ± 1.9	N/A	N/A
	SDMT	Attention	60.1 ± 17.4 (0.09)	57.4 ± 12 (0.04)	58.5 ± 12.6	N/A	N/A
	WLG	Execution	27.5 ± 5.6 (0.13)	27.4 ± 7 (0.17)	27.1 ± 7.4	N/A	N/A
	PASAT	Cognitive	75.6 ± 18.9 (−0.12)	46.3 ± 12.1 (−0.05)	49.3 ± 11.8	N/A	N/A

Mood and fatigue	HAD-A	Anxiety	6.7 ± 4.2	6.5 ± 3.6	5.2 ± 3.2	ns	ns
	HAD-D	Depression	3.1 ± 2.3	2.32 ± 2.12	1.35 ± 1.5	*p* = 0.005	*p* = *0.01*
	FSMC Cognitive	Cognitive fatigue	23.7 ± 9	22.7 ± 9.6	16.9 ± 6	*p* = *0.01*	*p* = *0.04*
	FSMC Motor	Motor fatigue	23.2 ± 10.4	23 ± 9.1	14.8 ± 5.8	*p* = 0.02	*p* = *0.001*

Disability and motor function	EDSS	General disability	1.6 ± 0.25 median: 1.5	1.7 ± 0.4	N/A	N/A	N/A
	TWT	Motor	0.2 ± 0.06 (−0.42)	0.3 ± 0.04 (0.63)	0.28 ± 0.04	N/A	N/A
	9HPT	Motor	0.05 ± 0.01 (−0.7)	0.05 ± 0.01 (−0.35)	0.06 ± 0.01	N/A	N/A

*Data are presented as mean raw scores ± SD; z-scores for continuous variables in patients are reported in brackets*.

*BRB-N, brief repeatable battery of neuropsychological tests; SRT-CLTR, selective reminding test-consistent long-term retrieval; SRT-recall, selective reminding test-long-term storage; SRT-delayed recall, selective reminding test-delayed recall; SDMT, symbol digit modalities test; PASAT, paced auditory serial addition test at 3 s; WLG, word list generation; HAD-A, Hospital Anxiety and Depression Scale-Anxiety; HAD-D, Hospital Anxiety and Depression scale-Depression; FSMC, Fatigue Scale for Motor and Cognitive functions; EDSS, Expanded Disability Status Scale; TWT, Timed 25-Foot Walk; 9HPT, Nine-Hole Peg Test*.

### Magnetic Resonance Imaging

**Figure 1:**
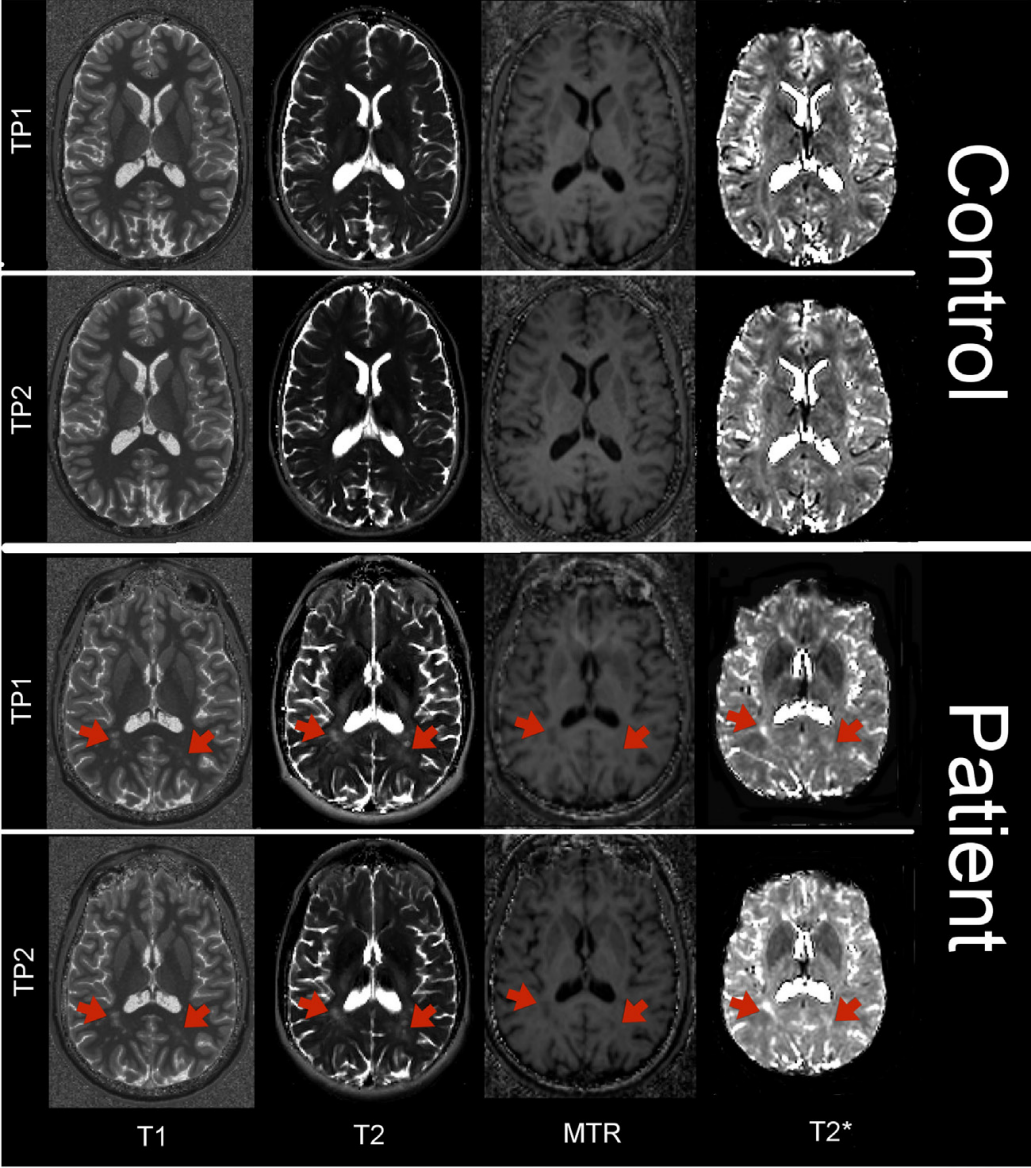
Axial view of T1 map, T2 map, T2* map, and MTR in a control and in a patient at time-point 1 and 2. Red arrows point at the location of MS lesions.

At both time-points, all subjects underwent a MRI protocol, including relaxometry, magnetization transfer, and structural MRI, as previously performed (26–28) and reported in detail in Table 3. T2* maps were obtained using an in-house correction method based on an estimated B1 field map, and MTR maps were computed using the following formula: MTR = (M0 − MT)/M0 where M0 and MT are the images acquired without and with MT pulse, respectively. Total scan time for T1, T2, T2* relaxometry, and MTR was ~23 min. Figure 1 shows an example of T1, T2, T2*, and MTR maps of a control and a patient at time-point 1 and 2.

**Table 3:** Magnetic resonance imaging protocol.

Sequence	TR/TE (TI) (ms)	Spatial resolution (mm^3^)	FoV (mm^3^)	AT (min:s)	Measurements
MP2RAGE	5,000/2.89 (700/2,500)	1.0 × 1.0 × 1.2	256 × 240 × 176	8:22	T1 map/lesion count and manual segmentation
T2*_M0/MT	1.23/47 (700/2,500)	1.6 × 1.6 × 1.6	256 × 240 × 160	5:38 (×2)	MTR/T2* maps
Multi-echo T2	5,000/9 TE1, 21 echoes	1.1 × 1.1 ×4.0	256 × 240 × 160	3:15	T2 map
MPRAGE	2,300/2.98 (900)	1.0 × 1.0 × 1.2	256 × 240 × 160	5:12	Structural (segmentation)
3DFLAIR	5,000/3,948 (1,800)	1.0 × 1.0 × 1.2	256 × 240 × 176	6:27	Lesion count and manual segmentation
DIR	10,000/218 (3,650, 450)	1.0 × 1.0 × 1.2	256 × 240 × 160	12:52	Lesion count and manual segmentation

*Data are presented as mean (SD) for continuous variables and count for categorical variables*.

*TE, echo time; TR, repetition time; TI, inversion time; FoV, field of view; AT, acquisition time*.

### MRI Post-Processing and Lesions Segmentation

For each subject, all images were registered in the MP2RAGE space using Elastix c++ library (33). Morphobox (34) was used to segment tissues and regions of interest (ROIs) on MPRAGE images for both time-points. The following ROIs were automatically segmented at both time-points: white matter, cortical gray matter, thalamus, and basal ganglia (caudate, putamen, and globus pallidus). Lesions were manually segmented by consensus on 3D FLAIR, DIR, and MP2RAGE images at both time-points by a radiologist (5 years of experience) and a neurologist (10 years of experience), as previously performed (26, 27). MS lesions were also manually classified as WM, GM, and mixed WM/GM lesions. Normal-appearing (NA) tissue was obtained in each ROI by subtracting the lesions mask. Lobar segmentation was obtained using Morphobox (34). Two-timepoint percentage brain volume change (PBVC) was estimated using Structural Image Evaluation, using Normalisation of Atrophy (35), which is part of FSL https://fsl.fmrib.ox.ac.uk/fsl/fslwiki. All volumetric measurements were normalized by the total intracranial volume.

### Lesions *z*-Scores and Lesions Classification

In the all cohort of 18 healthy controls, we calculated the mean T1, T2, and T2* rt and MTR in the gray and white matter components of the segmented ROIs. Then, we calculated a *z*-score for each lesion for all parametric maps at both time-points to quantify the deviation of each specific MR metric (e.g., T1) in MS lesions from the distribution of the same signal in the corresponding healthy tissue in controls:

$$Z_{\text{T}1}=\frac{1}{N}\sum_{v\in l}^{l}\frac{I_{\text{T}1}(v)-{\text{μ}}_{{\;}_{\text{T}1}}(L_{l},{\text{T}}_{l})}{{\text{σ}}_{\text{T}1}(L_{l},{\text{T}}_{l})}$$

where *Z*_T1_ corresponds to the lesion *z*-score, *v* to the lesion voxels, *N* to a normalization term, *I*_T1_ to the T1 quantitative map, μ_T1_ to the mean, and σ_T1_ the SD of the T1 relaxation in the lobe *L_l_* and tissue *T_l_* (i.e., WM or GM) in the controls group, corresponding to the lesion location and type. We considered the distribution in the healthy tissue corresponding to the lobe where lesions are located because previous studies reported lobar variations of qMRI parameters (36, 37).

WM, GM, and mixed WM/GM lesions were classified in four classes according to the evolution of their volume across time-points as previously reported (38): (i) stable, (ii) enlarged (increase of at least 50% of lesion volume between time-points), (iii) shrunken (decrease of at least 50% of lesion volume between time-points), and (iv) resolved.

### Statistical Analysis

#### Cross-sectional Analysis between Patients and Controls at Baseline

##### Clinical Scores

All patients’ neurocognitive scores were standardized using *z*-scores computed from mean and SD of 18 HC clinical scores. Behavioral scores and disability scores were compared between patients and controls at baseline and follow-up using a student *t*-test.

##### qMRI in Lesions and NA Tissue

An analysis of variance (ANOVA) with age as covariate was performed to compared differences between mean T1, T2, T2*, and MTR in lesions in MS patients to healthy tissue in controls at baseline. ANOVA with age as covariate was also used to assess the presence of differences at baseline between mean T1, T2, T2*, and MTR in NAWM, NAGM, and basal ganglia tissue in patients vs controls.

#### Longitudinal Analysis in Patients and Controls

##### qMRI Evolution in NA Tissue

First, we performed a global omnibus test (*F*-test) to assess the interaction between group (patients and controls) and timepoint.

Then, we performed paired repeated measures ANOVA with age and gender as covariate in the patients and control group in order to assess the following H0 hypotheses:

H0-1:there are no differences in the mean T1, T2, T2*, MTR in NAGM and NAWM between TP1 and TP2.
H0-2:there are no differences in the mean T1, T2, T2*, MTR in the thalamus, and basal ganglia (caudate, putamen, and pallidus) between TP1 and TP2.

Correction for multiple comparisons was performed using Bonferroni (H0-1:2 regions, 4 contrasts = 8 tests; H0-2:4 regions, 4 contrasts = 16 tests).

##### Brain Volume Evolution

To assess differences in PBVC between time-points in patients and controls, we used an ANOVA with age and gender as covariate.

##### qMRI *z*-Scores and Volume Evolution in Lesions

We analyzed the changes in lesions *z*-scores and lesion volumes by performing a paired *t*-test between TP1 and at TP2. To assess changes in lesions *z*-scores, we calculated T1, T2, T2*, and MTR *z*-scores in the TP1 lesion mask and the same region in TP2 images. Paired *t*-tests were applied separately to WM, GM, and mixed lesions to evaluate whether qMRI parameters evolved differently across different lesion types. Correction for multiple comparisons was performed using Bonferroni (3 lesion types, 4 contrasts = 12 tests).

##### Analysis of Tp1 qMRI Metrics in Lesions with Different Volumetric Evolution at Follow-up

We performed ANOVA to compare the mean T1, T2, T2*, and MTR *z*-scores at TP1 across lesions with different volumetric evolution (i.e., stable, enlarged, shrunken, resolved). ANOVA were applied separately to WM, GM, and mixed lesions. We also combined the ANOVA with Tukey tests to find which lesion class was significantly different from the others. Correction for multiple comparisons was performed using Bonferroni (3 lesion types, 4 contrasts = 12 tests).

##### Longitudinal Analysis of Clinical Scores in Patients

We evaluated the evolution of clinical scores by performing paired *t*-test between patients clinical scores (*z*-cores for continues variables and raw scores for the others) at TP1 and at TP2.

#### Regression Analysis between qMRI and Clinical Scores Evolution

A multivariate linear regression of clinical scores evolution was performed using a general linear model with qMRI measurements that significantly evolved over time as regressors and clinical *z*-scores/test scores that significantly changed between time-points as predicted variables. Based on the results of the longitudinal analysis of NA tissue and lesions, we selected the following regressors: (i) T2 mean variation between time-points in NAWM; (ii) mean T1, T2, T2*, and MTR lesions *z*-score variation between time-points; and (iii) mean lesions volume variations between time-points. Though the lesions volume did not show significant changes between time-points, we added it to compare its contribution to the regression with qMRI measurements. Age and gender were used as covariates. Clinical *z*-scores were adapted using Box–Cox transformation to satisfy model assumption for normality.

We used a backward stepwise approach to select the best prediction model for each dependent variable (clinical *z*-scores/tests evolution). Bonferroni correction was applied for multiple comparisons (3 clinical *z*-scores evolution tested). “Leave-one-out” cross validation (LOOV) was used to assess the prediction quality and robustness of each model.

All regression analyses were performed using R software (http://www.R-project.org).

## Results

### Cross-sectional Analysis between Patients and Controls at Baseline

At baseline, we measured a highly significant difference between lesions and healthy tissue for all qMRI metrics (*p* < 1e−10). Also, at baseline, no significant differences were observed between mean qMRI metrics in NAWM, NAGM, and basal ganglia in patients vs controls.

Differences in cognitive scores at baseline (TP1) and follow-up (TP2) were reported as *z*-scores in Table 2. As to behavioral scores, at baseline MS patients exhibited a higher depression (*p* = 0.005) and cognitive (*p* = 0.01) and motor (*p* = 0.02) fatigue compared to healthy subjects. At follow-up, MS patients had still higher depression (*p* = 0.01) and cognitive (*p* = 0.04) and motor (*p* = 0.001) fatigue compared to healthy subjects.

### Longitudinal Analysis in Patients and Controls

#### qMRI Evolution in NA Tissue

**Figure 2:**
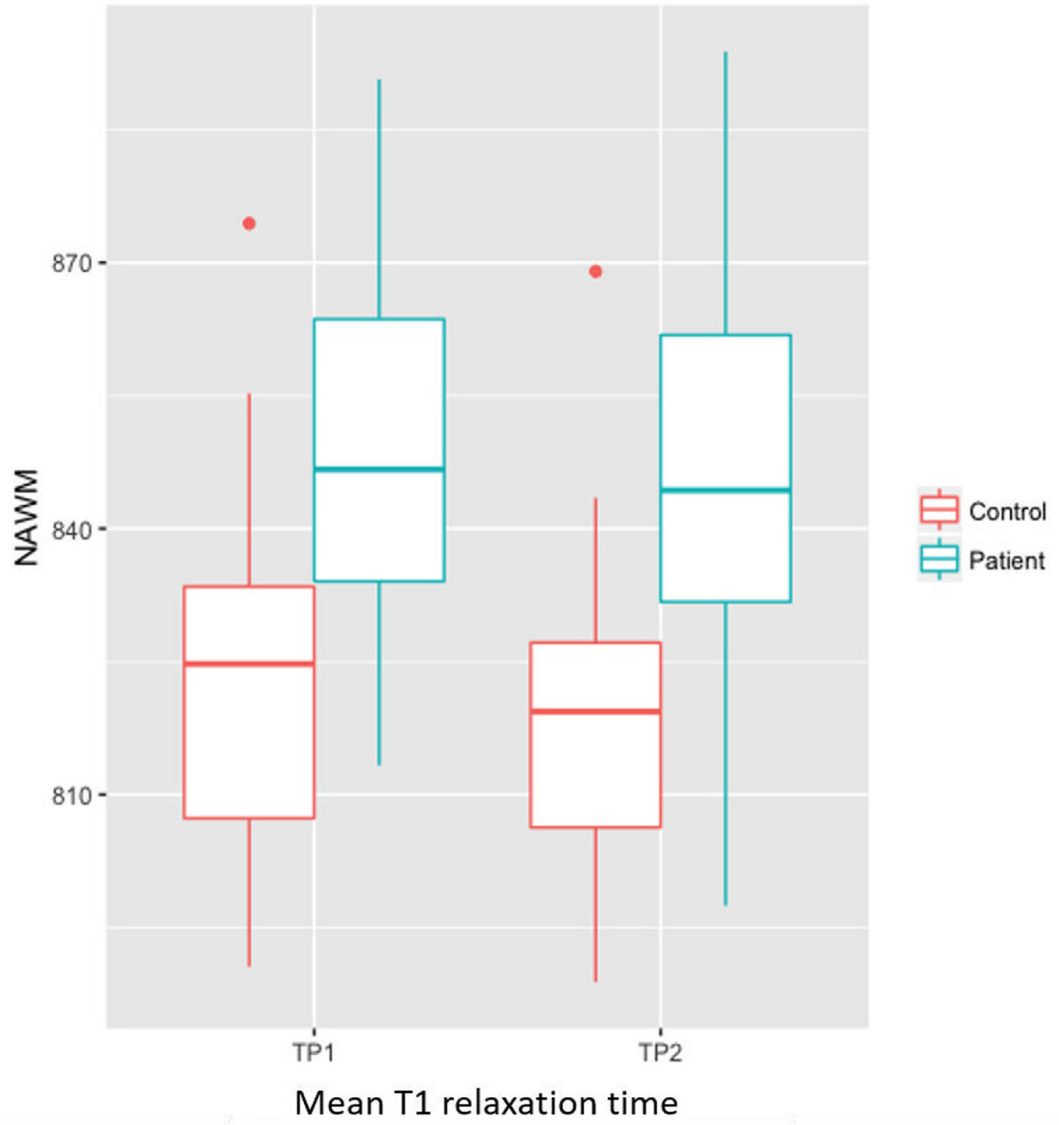
Bar plot showing significant higher T1 in NAWM of patients compared to controls at both time-point (*p* = 0.02).

The *F*-test did not show any significant interaction between group (patients/controls) and time-points (time-point 1 and 2). The *F*-test also revealed a significant higher T1 in patients compared to controls at both time-point (*p* = 0.02), Figure 2.

**Figure 3:**
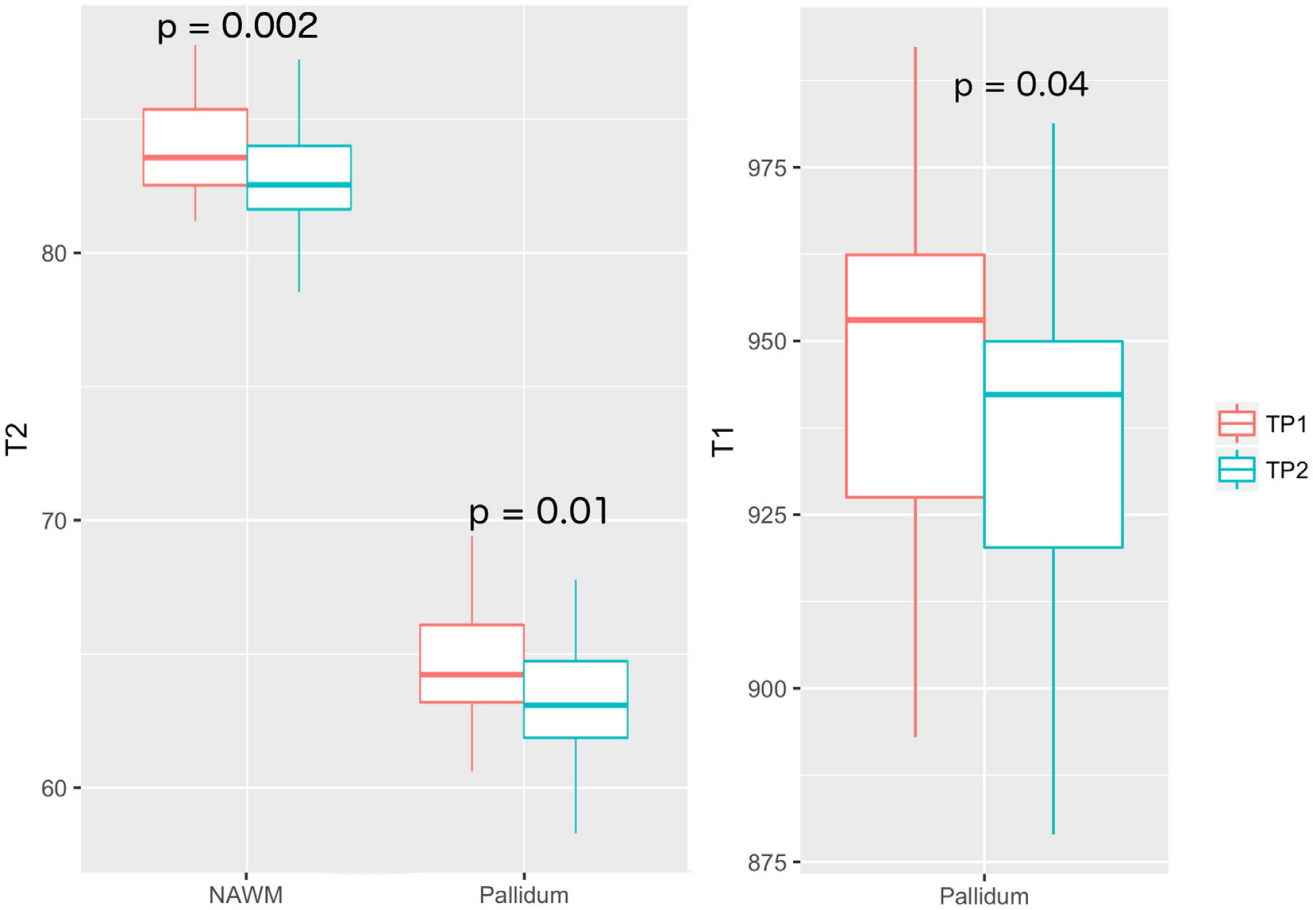
Mean T2 relaxation times in NAWM and pallidum of MS patients, at both time-points (left). Mean T1 relaxation times in pallidum of MS patients (right).

In patients, we measured a significant longitudinal decrease in T2 relaxation times in NAWM (*p* = 0.002, T2_TP1_ = 83.4 ± 5.6 ms, T2_TP2_ = 82.6 ± 5.3 ms), thalamus (*p* = 0.01, T2_TP1_ = 89.2 ± 4.4 ms, T2_TP2_ = 87.1 ± 4.8 ms), pallidum (*p* = 0.01, T2_TP1_ = 64.7 ± 6 ms, T2_TP2_ = 63.6 ± 5 ms) and putamen (*p* = 0.0004, T2_TP1_ = 75.4 ± 2.7 ms, T2_TP2_ = 74.2 ± 2.5 ms), and a decrease in T1 relaxation times in the pallidum (*p* = 0.04, T1_TP1_ = 945 ± 883 ms, T1_TP2_ = 935 ± 1020 ms), Table 4 and Figure 3. No longitudinal differences were observed in the control group (NAWM: T2_TP1_ = 82.4 ms, T2_TP2_ = 82.0 ms; pallidum: T2_TP1_ = 64.6 ms, T2_TP2_ = 63.7 ms; T1_TP1_ = 931 ms, T1_TP2_ = 913 ms).

**Table 4:** Longitudinal analysis results of patients.

Regions of interest	T1		T2		MTR	
	*p*-Value	CI (95%)	*p*-Value	CI (95%)	*p*-Value	CI (95%)
**Normal-appearing tissue**						

NAWM	n.s		0.002* (83.4, 82.6)	[0.4–1.3]	n.s	
NAGM	n.s		n.s	N.A	n.s	

**DGMN**						

Thalamus	n.s		0.01* (89.2, 87.1)	[0.9–3.2]	n.s	
Pallidum	0.04* (945, 935)	[3.6–15.3]	0.01* (64.7, 63.6)	[0.5–1.7]	n.s	
Putamen	n.s		0.0004** (75.4, 74.2)	[0.7–1.6]	n.s	
Caudate	n.s		n.s	N.A	n.s	

**Lesions (*z*-scores)**						

WM	2.2e−14*** (6.3, 5.8)	[0.7–0.9]	n.s		9.07e−03* (−1.4, −1.3)	[−0.2 to 0.06]
GM	n.s		n.s		n.s	
Mixed	3e−03** (13.1, 12.2)	[0.3–1]	n.s		0.03* (−2.8, −2.4)	[−0.3 to 0.07]

*p < 0.05, **p < 0.001, ***p < 0.00001, corrected p-values are reported when significant. T1, T2, T2*, and MTR mean values and z-score in MS lesions at TP1 and TP2 are reported in brackets.

*CI, confidence interval; HSD, Honest Significant Difference*.

#### Brain Volume Evolution

No significant changes in PBVC were observed between patients and controls over 2 years (*p* = 0.4).

#### qMRI *z*-Scores and Volume Evolution in Lesions

MS lesions showed a global decrease in MTR and increase in T1 (*p* = 8e−6 and *p* < 1e−15).

Seventy-five percent (75%) of the lesions were WM lesions (548 lesions), 22% were mixed WM/GM lesions (160 lesions), and 3% were GM lesions (25 lesions).

**Figure 4:**
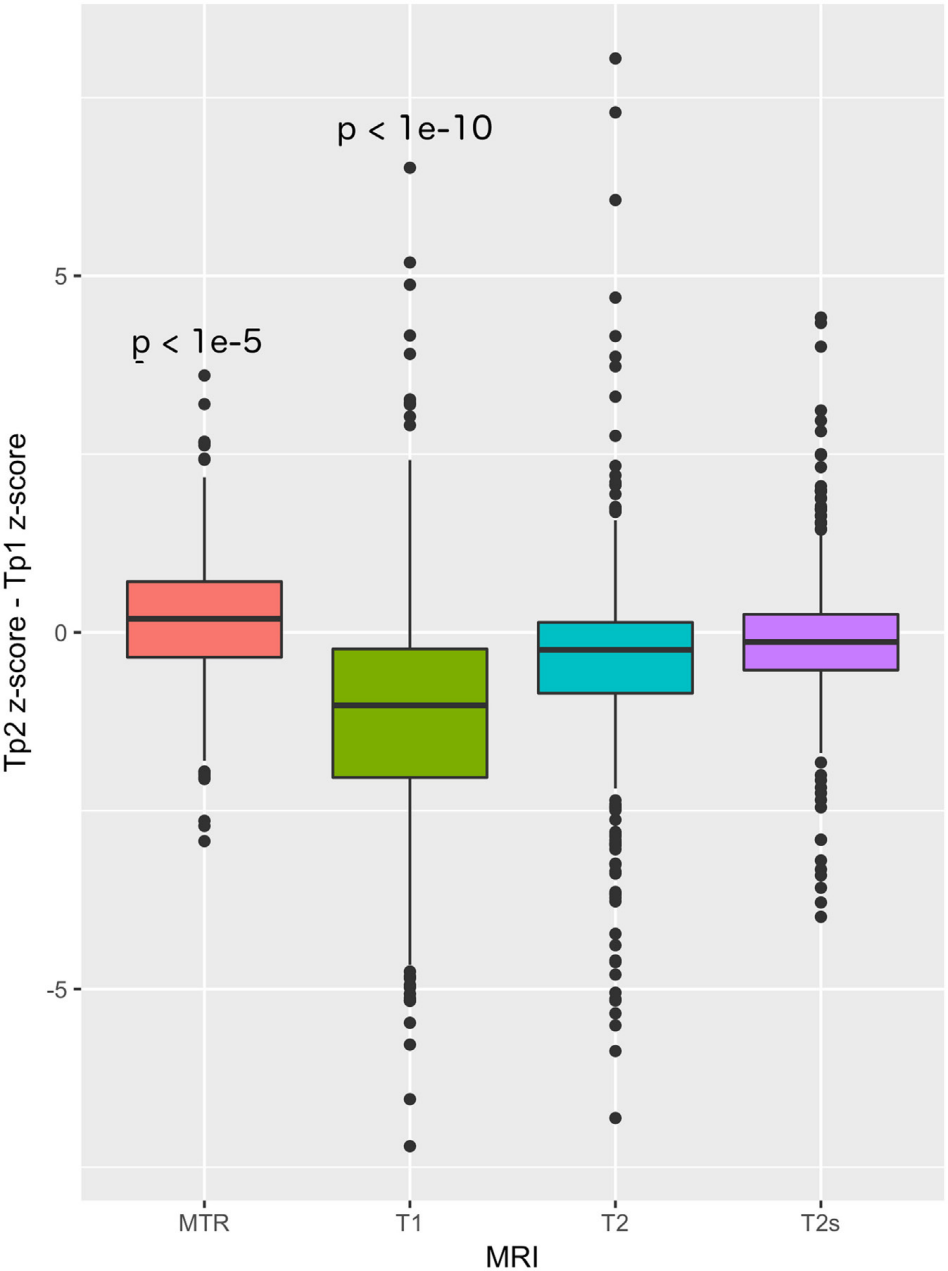
T1, T2, T2*, and MTR *z*-scores differences between baseline and follow-up in MS lesions.

In both WM and mixed WM/GM lesions, we found significant changes in T1 and MTR *z*-scores between time-points. WM lesions showed a significant decrease in T1 *z*-scores (*p*-value < 1e−10, *z*_TP1_ = 6.3 ± 1.5, *z*_TP2_ = 5.8 ± 1.6), as well as a significant increase in MTR *z*-score (*p*-value = 9.07e−3, *z*_TP1_ = −1.4 ± 0.5, *z*_TP2_ = −1.3 ± 0.5). Similarly, mixed lesions showed a significant decrease in T1 *z*-score (*p*-value = 3e−3, *z*_TP1_ = 13.1 ± 5.8, *z*_TP2_ = 12.2 ± 6.4) and an increase of MTR *z*-score (*p*-value = 0.03, *z*_TP1_ = −2.8 ± 1.4, *z*_TP2_ = −2.4 ± 1.6), Table 4 and Figure 4.

#### Analysis of qMRI at Tp1 in Lesions with Different Volumetric Evolution

Over 2 years follow-up, 75% of lesions remained stable (548 lesions), 13% were enlarged (99 lesions), 1% was shrunken (8 lesions), and 11% were resolved (78 lesions).

At TP1, we measured a significant difference in T1 (*p* = 0.024), T2 (p = 0.004), T2* (*p* = 0.0012), and MTR (*p* = 0.01) *z*-scores between stable, shrunken, resolved, and enlarged WM lesions.

**Figure 5:**
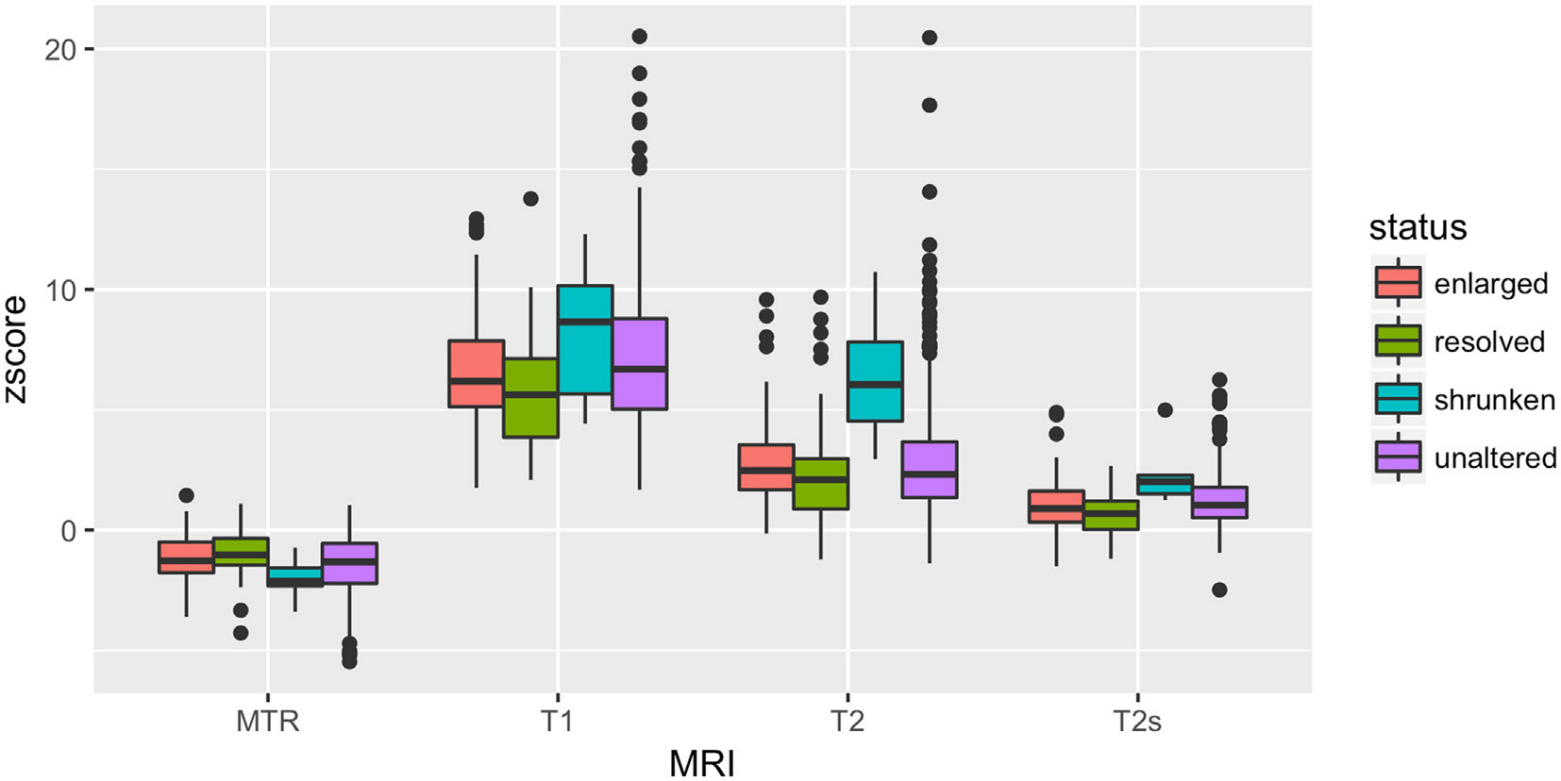
At baseline, T1, T2, T2* and MTR z-scores differences among stable, enlarged shrunken and resolved WM lesions.

At TP1, WM resolved lesions showed a significantly lower T1 (*p* = 0.005) and T2* (*p* = 0.002) *z*-scores and a higher MTR *z*-score (*p* = 0.007) compared to WM stable lesions. WM shrunken lesions exhibited a significantly higher T2 *z*-scores compared to WM stable (*p* = 0.0003), enlarged (*p* = 0.0004) and resolved lesions (*p* = 0.0001), Table 5 and Figure 5.

**Table 5:** Analysis of variance (ANOVA) and Tukey HSD test on Tp1 QMRI *z*-scores across lesions groups.

WM Lesions	ANOVA	Tukey HSD			
	*p*-Value	Group comparison	Mean difference	CI (95%)	Adjusted *p*-Value
T1 *z*-score	0.024*	Stable-resolved	1.3	[0.3, 2.3]	0.005*

T2 *z*-score	4.2E−03*	Shrunken-enlarged	3.6	[1.2, 5.9]	0.0004**
		Shrunken-resolved	3.9	[1.6, 6.3]	0.0001**
		Shrunken-stable	3.5	[1.2, 5.7]	0.0003**

T2* *z*-score	0.0012*	Shrunken-resolved	1.6	[0.5, 2.6]	0.001*
		Stable-resolved	0.5	[0.1, 0.9]	0.002*

MTR *z*-score	0.013*	Stable-resolved	−0.5	[−0.9, −0.1]	0.007*

*p < 0.05.

**p < 0.001.

***p < 0.00001.

*CI, confidence interval; HSD, Honest Significant Difference*.

No differences were found for GM and mixed lesions.

#### Longitudinal Analysis of Clinical Scores in Patients

At time-point 2 compared to time-point 1, we measured a significant decrease in (i) MSFC Timed 25-foot Walk-TWT (*p* = 5.838e−05), as well as a significant increase in (i) selective reminding test—long-term storage (SRT-CLTR, *p* = 0.04), and an increase in (ii) t selective reminding test—consistent long-term retrieval (SRT-LTS, *p* = 0.02) *z*-scores.

### Regression Analysis between qMRI and Clinical Scores Evolution

**Figure 6:**
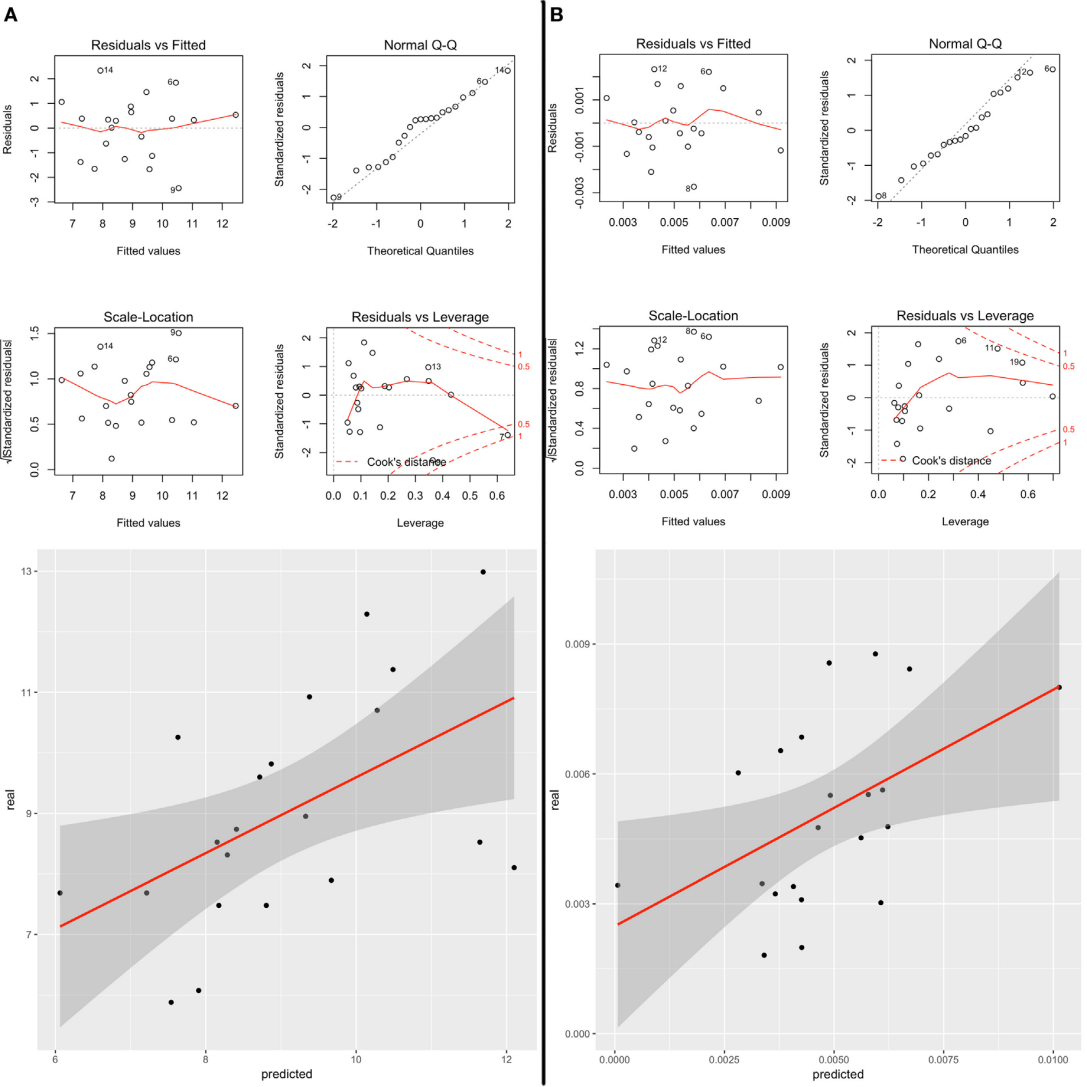
**(A)** Correlation between predicted and real measurements of (A) SRT-CLTR *z*-score and (B) TWT after leave-one-out cross validation. **(B)** Diagnostic plots shows: (i) residuals function of fitted values (upper left), (ii) the hypothesis of normal distribution of the errors with QQplot (upper right), (iii) that the variance in the Residuals does not change as a function of *z*-score (bottom left), (iv) and the presence of outliers with Residuals function of Leverage (bottom right).

General linear model using stepwise regression revealed strong associations between qMRI features and clinical scores variations between time-points, confirmed by a leave-one-out cross validation (i) baseline T2, T2*, and MTR *z*-scores variations in lesions and T2 variation in NAWM were significantly correlated with changes in deambulation (MSFC-TWT score, adjusted *R*^2^: 0.49, *p* = 0.013; LOO Spearman correlation score: 0.5), Figure 6A; and (ii) baseline T1, T2, and T2* *z*-scores variations in lesions were significantly correlated with changes in selective reminding test—long-term storage (BRB-N-CLTR *z*-score, adjusted *R*^2^: 0.48, *p* = 0.007; LOO Spearman correlation score: 0.52), Figure 6B.

## Discussion

Our study shows that the combination of multiple qMRI metrics is sensitive to 2-year changes in brain pathology in a group of early-stage MS patients on therapy, suggesting a decrease in NAWM inflammation as well as repair processes in lesions. Besides, our work provides evidence that multiple qMRI metrics at baseline are strongly and differently related to patients’ motor and cognitive function at two year follow-up.

To date, there are very few studies that combined qMRI metrics to study the evolution of MS pathology over time and none applied T1, T2, T2* relaxometry, and MTR. A small number of longitudinal studies in MS patients applied either MTR (39–42) or T2* relaxometry maps (43). These works showed a progressive increase in MTR over time in subgroups of lesions [i.e., nearly 20% of acute lesions according to Chen et al. (40)] and a decrease in T2* rt in the basal ganglia (43) in MS patients, who were followed-up between 1 and 4 years. Changes in MTR and T2* rt were interpreted as remyelination in lesions and iron accumulation in basal ganglia; however, these interpretations may have been misleading because an increase in MTR and a decrease in T2* might be also due to a decrease in water content, i.e., edema reabsorption (20, 26).

Recent developments in MRI technology have made it possible to acquire multiple advanced MRI contrasts in scan times compatible with clinical research, thereby increasing sensitivity and specificity to brain pathology (21, 26, 27, 44). By combining multiple MR metrics and leveraging their biophysical properties, we have previously studied the pathology of MS lesions, NA tissue and brain connectivity (26, 28) and proposed a new classification of MS lesions (27). Here, we have developed an automated qMRI framework to monitor disease evolution in early MS patients.

Despite the absence of volumetric changes in the majority of lesions (75%), qMRI showed a concomitant significant decrease in T1 relaxation times and an increase of MTR suggesting repair mechanisms such as remyelination, increase in axonal density and/or gliosis (18, 45). Future studies combining qMRI with diffusion MRI may allow discriminating between gliotic or axonal/myelin restauration by exploiting the information on tissue anisotropy provided by the diffusion contrast.

The few lesions that disappeared at 2-year follow-up (resolved lesions) exhibited higher MTR and lower T1 and T2* rt at baseline than lesions that did not change in volume. While higher MTR may indicate at the time higher free-water content (i.e., more pronounced edema due to active inflammation) or higher higher myelin/axonal bound water (i.e., higher myelination/axonal density), the concomitant lower T1/T2* rt point at the latter pathophysiological explanation. In fact, the presence of edema would have increased—not decreased—both T1 and T2* rt (6).

Also, lesions that significantly decreased in volume at 2-year follow-up (shrunken lesions), evidenced a significant higher T2 rt at baseline compared to stable and other lesions, possibly related to higher inflammatory processes (i.e., high presence of extracellular water) or higher cellular infiltration (i.e., lower intracellular water). The absence of significant differences at baseline between lesions that enlarge at follow-up and other lesions suggests that the lesion microstructural properties as measured by qMRI is not predictive of lesion growth in this cohort of patients.

These findings provide new evidence that qMRI may be used as baseline biomarker of lesion evolution and extend current literature reporting that lesions with modest MTR decrease compared to healthy tissue are more likely to undergo partial or complete recovery (18, 46).

The study of NA tissue revealed a mild but significant longitudinal decrease in T2 rt in NAWM and a decrease of both T2 and T1 rt in the pallidum, whereas no longitudinal changes were observed in healthy subjects for all aMRI parameters. On one hand, these data provide evidence of the stability of qMRI in control subjects. On the other hand, our results suggest that—in our cohort of early MS patients with minimal clinical deficits—there is a longitudinal decrease in overall inflammation, i.e., due to lower microglia activation in patients on therapy (47) and/or iron accumulation (16). Overall these findings suggest that qMRI metrics may help defining the underpinnings of disease evolution in MS patients and provide new biomarkers of disease impact over time in the absence of significant brain volume changes.

Last, lesions and NAWM characteristics, as measured with qMRI, appeared to be strongly related to motor and cognitive changes at 2-year follow-up, whereas lesion volume did not. These results extend the evidence obtained in a previous cross-sectional study from our group, where a number of cognitive functions exhibited high correlations with lesion and NA tissue properties measured with qMRI (26). MS patients improved their motor and cognitive function over 2 years: while on one hand, cognitive improvement may be attributed to learning despite we adopted an adapted test for longitudinal assessment, the presence of both cognitive and motor improvement in a very homogeneous cohort of early-stage RRMS patients on therapy may well indicate compensatory plasticity.

The study suffers from some limitations. First, we studied a cohort of patients and healthy controls of moderate/small size. Future study should aim at confirming our findings in larger populations and extend them to more advanced disease stages. Second, since we studied a cohort of early-stage MS patients, we do not have histological data to corroborate our findings; therefore, we interpreted our results based upon the biophysical properties of the MR signals (6) and upon a number of previous publications using single contrasts parametric MRI and histopathology [for review see Ref. (48)]. However, though histological data might be valuable to assess changes in myelin and axons, *postmortem* experiments in patients will not provide any direct information about the presence of extracellular water accumulation (i.e., edema), as we do in the current study. To note, the only one study that—to our knowledge—attempted at correlating *ex vivo* T1/T2 relaxation times and MTR with histopathological metrics evinced the limits of *ex vivo* MRI to measure the complexity of phenomena influencing the MRI signal *in vivo* (49). Last, another potential limitation of our work is that the applied T2 relaxometry maps had a lower resolution than the other maps, which could have decreased the sensitivity to lesions and NA tissue pathology.

In summary, we have shown that combining different MR parameters allows increasing pathological specificity to ongoing damage even at early MS stages and provide metrics that predict patients motor and cognitive function at 2 years follow-up. In the future, we will assess the sensitivity of qMRI metrics to larger and more heterogeneous patients cohorts, we will establish the sensitivity of qMRI metrics to different therapy regimens as well as work on the development of “personalized” qMRI assessments.

## Ethics Statement

The study was approved by the Ethic Committee of the Lausanne University Hospital (CHUV); all participants gave written informed consent prior to participation.

## Author Contributions

Study design (CG and GK); collection, analysis, and interpretation of data (GB, BM, MF, JM, MS, GK, CG, and PF); writing of the report and decision to submit the paper for publication (GB, BM, JM, SS, MS, RP, J-PT, GK, and CG).

## Conflict of Interest Statement

GK and BM are Siemens Healthcare employees. The other authors have nothing to disclose. The reviewer EP and handling editor declared their shared affiliation, and the handling editor states that the process nevertheless met the standards of a fair and objective review.
